# Haematopoietic Traits in Indigenous and Modern Pig Breeds Are Modulated by Housing System and Developmental Stage

**DOI:** 10.1002/age.70103

**Published:** 2026-04-16

**Authors:** Kanishka Kapoor, Michael Oster, Henry Reyer, Cornelia C. Metges, Eduard Muráni, Siriluck Ponsuksili, Klaus Wimmers

**Affiliations:** ^1^ Research Institute for Farm Animal Biology (FBN) Dummerstorf Germany; ^2^ Chair of Animal Breeding and Genetics, Faculty of Agricultural, Civil and Environmental Engineering University Rostock Rostock Germany

**Keywords:** Haematopoiesis, immune resilience, organic farming, pig welfare, traditional breed

## Abstract

Haematological parameters are important indicators of health, physiological status and resilience. In pigs, these parameters are influenced by both exogenous factors, such as feeding, housing and weaning age and intrinsic factors, including physiological maturity and genetic background. In this study, complete blood counts from 512 German Landrace (GL) and German Saddleback (GS) piglets kept in conventional (CON) and organic (ORG) housing systems were analysed throughout four developmental stages until day 70 of life. The GS consistently showed significantly higher counts of red and white blood cells, alongside higher counts of leukocyte subsets such as lymphocytes and monocytes, indicating a potentially efficient immune profile compared to GL. In contrast, GL exhibited larger erythrocytes with higher haemoglobin content as reflected via higher MCV and MCH levels, potentially endowing GL pigs to cope with the high metabolic demands for growth and performance. Housing effects were observed at day 70, where pigs kept in ORG showed reduced HCT and MCV, but higher MCHC levels, which might be attributed to feed components in ORG husbandry, potentially reflecting dietary limitations in methionine. Notably, platelet counts were higher in GL compared to GS piglets under ORG conditions, implying a breed‐dependent haematopoietic response. In summary, these findings indicate a trade‐off between erythrocyte quantity and haemoglobin‐loading capacity, with potential implications for breed‐specific myoglobin characteristics. The leukocytes profile further suggests breed‐related differences in immune resilience which need to be validated by functional analyses to inform housing and management strategies for weaning in both modern and indigenous pig breeds.

## 1 Introduction

Intensive pig farming practices, associated with increased productivity, have been shown to be linked to concerns about animal welfare, environmental burdens, and veterinarian interventions (Ballester et al. 2020). Public awareness regarding pig welfare has increased for matters such as space allowance, freedom of movement, expression of natural behaviours, timing of separation of offspring from the sows, and the subsequent associated stress responses (Ahsan Kabir 2019; Delsart et al. 2020; Motta et al. 2019).

Physiologically, weaning is recognised to be one of the most stressful events in a pig's life affecting health and immunity (Gimsa et al. 2018) primarily due to maternal separation, social reorganisation, sudden dietary changes (from sow's milk to solid feed) and increased exposure to environmental pathogens (Bhattarai and Nielsen 2015; Buchet et al. 2017; Campbell et al. 2013; Tang et al. 2022). Management strategies such as increasing weaning age, housing systems used during parturition and early neonatal adaptation, gradual separation from the mother through intermittent suckling, co‐mingling of litters, and providing creep feed have been debated in terms of their potential to reduce stress and improve gastrointestinal function and immune competence (Lickfett et al. 2025a, 2025b; Van Kerschaver et al. 2023). A delayed weaning may offer some immunological benefits, which can be investigated via haematological parameters, e.g., complete blood counts, and offer valuable insights into the piglets' physiological and health status (Jelek et al. 2018; Oven et al. 2021). For example, it has previously been shown that various haematological components, particularly the counts and ratios of immunocompetent cell types like lymphocytes and neutrophils, can serve as biomarkers of immune resilience in pigs (Bai et al. 2020; Ballester et al. 2020; Dervishi et al. 2021). Studies in suckling crossbred piglets have shown detailed developmental increases in circulating T and B lymphocyte populations during the first weeks of life (Harayama et al. 2022). Passive immunity is acquired via immune components from the sow's colostrum and milk (Bandrick et al. 2014; Li et al. 2024), however, this maternal protection significantly declines by 4–6 weeks of age (Martínez‐Boixaderas et al. 2022), a period commonly associated with weaning. This decline coincides with the piglet's own developing immune system towards a long‐term disease resistance (Diehl et al. 2022).

Conventional husbandry systems employ practices that have led to new EU Regulations (EC No 834/2007) that call for environmentally responsible farming approaches, diversity, and sustainability, such as organic husbandry systems. Conventional systems generally use climate‐controlled indoor facilities, high stocking densities and slatted flooring, which provide highly standardised conditions for the full exploitation of the genetic potential (Hämeenoja 2002). Additionally, a key management practice in many conventional systems is early weaning of piglets, usually between 21 and 28 days of life. On the other hand, in organic pig husbandry systems stocking densities are lower; the animals have access to outdoor space, straw bedding, organic feed and piglets must be weaned at a minimum age of 40 days (von‐Borell et al. 2001). Evidence suggests that extensive and enriched environments can enhance immune function and stress adaptation (Millet et al. 2005; Nirea and Meuwissen 2017).

It is assumed that the breed component is relevant for adaptation and performance in the respective housing environment. The German Landrace (GL) is a modern, high‐performing breed characterised by rapid growth, high fertility and lean‐meat content (Falkenberg and Hammer 2006; Rehfeldt et al. 2012). In contrast, the German Saddleback (GS) is a non‐commercial, indigenous breed which has greater fat deposition and relatively lower litter sizes (Giovannini et al. 2023; Lickfett et al. 2025a; Olschewsky et al. 2026). In fact, pigs from the two breeds differ fundamentally in terms of metabolism, performance, body composition, and breeding history (Nürnberg 1997; Oster et al. 2025; Ponsuksili et al. 2024).

The distinct breed characteristics and husbandry systems provide insights into the adaptability and resilience of breeds to various husbandry environments. Animals better suited to high‐input conditions like GL may not perform optimally in lower‐input environments, such as those conditions found in organic housing. In contrast, indigenous breeds like the GS might be attributed to a greater adaptability to less controlled environments, showing enhanced immune resilience and metabolic adaptability (Song et al. 2020; Wang et al. 2024).

In this study, it is hypothesised that pig breed and environmental factors determined by the housing system are reflected by haematological traits related to the red and white blood cell profiles in a defined pig population. This study investigated how indigenous and modern pig breeds, raised under conventional and organic husbandry systems, influence haematological parameters. Complete blood counts were evaluated at four time points, including suckling period, pre‐weaning, weaning and juvenile stages.

## 2 Materials and Methods

### 2.1 Ethics Statement

All experiments were performed in accordance with the German Animal Welfare Act, and EU Directive 2010/63/EU (Annex VIII, Section III). The study complied with the ARRIVE guidelines and received ethical approval from the Ethics Committee of the federal state of Mecklenburg‐Western Pomerania, Germany (LALLF M‐V/TSD/7221.3–1‐030/21).

### 2.2 Experimental Design and Animals

The study comprised repeated blood samples in piglets (*n* = 512) to investigate haematological parameters comprising two pig breeds, the modern German Landrace (GL) and the traditional, indigenous German Saddleback (GS) under two husbandry conditions, i.e., a conventional indoor system (CON) and an organic system (ORG) in a 2 × 2 design. Consequently, the study yielded four experimental groups of piglets, with GL‐CON (*n* = 155), GL‐ORG (*n* = 130), GS‐CON (*n* = 116), GS‐ORG (*n* = 111). The piglets were retrieved from a total of 40 sows originating from certified herd books, which were artificially inseminated with purebred boars. Sows were utilised during their 1st, 2nd and 3rd gestation (primiparous: *n* = 10; multiparous: *n* = 30). In total, the trial made use of 91 farrowings. Data were collected from 235 piglets born to first‐parity sows, 163 piglets born to second‐parity sows, and 114 piglets born to third‐parity sows. About 24–48 h after each farrowing, a fixed number of piglets balanced for sex (three males and three females) were selected for intensive monitoring and blood sampling. Piglets within the 10th and 90th percentile of birth weight in each litter were selected to exclude extremely small or large piglets.

### 2.3 Housing and Management

All animals were raised at the FBN experimental pig facilities under either CON or ORG. Housing systems differed in space allowance, management, feeding regimes and feed composition (Table S1). Animal care staff and daily routines were consistent between housing systems.

During gestation (until day 105), CON sows (*n* = 11 GL; *n* = 9 GS) were housed indoors (5.5 m^2^/sow) in three‐area design pens (partially slatted, straw‐bedded lying area, self‐locking feeding stalls) and received a commercial diet twice daily. ORG sows (*n* = 10 GL; *n* = 10 GS) were group‐housed in straw‐bedded pens (~2.6 m^2^/sow) with continuous outdoor access (~2.0 m^2^/sow) and were fed an organic, high‐fibre diet via an automated feeder station. The ORG system additionally offered wood chains and an automatic brush. *Ad libitum* water and a 12 h light/dark cycle were maintained for all gestating sows.

For farrowing and lactation (from day 105 of gestation), sows were moved to respective farrowing units. ORG sows farrowed in larger pens (~8.4 m^2^ indoor and ~5 m^2^ outdoor areas) with deep straw bedding, nesting material, and outdoor access for piglets, receiving an organic lactation diet *ad libitum*. CON sows were housed in conventional pens (~6.5 m^2^) with hinged crates restricting movement for approximately 5 days around farrowing. CON sows were hand‐fed a commercial lactation diet. Both systems included a heated creep area for piglets, supervised farrowings with obstetric support as needed, and nipple drinkers for sows and piglets.

Piglets were weaned at 28 days in the CON system and at 42 days in the ORG system. Post‐weaning, ORG piglets were moved into straw‐bedded nursery pens (~7 m^2^ indoor and 4.2 m^2^ outdoor areas) with heated areas. CON piglets were mixed into groups of ~10–15 animals and housed in indoor, climate‐controlled pens with partially slatted floors and limited bedding. All piglets received appropriate commercial (CON) or organic (ORG) solid pre‐starter diets from day 14 of life (Table S1) *ad libitum*. Respective starter diets were provided after weaning (Table S1). Routine health measures and biosecurity protocols (e.g., dedicated clothing, footbaths) were applied consistently in both systems. Piglets were vaccinated against *Lawsonia intracellularis* (Enterisol Ileitis; Boehringer Ingelheim Vetmedica GmbH, Ingelheim, Germany) one week before pre‐weaning (age day 21 of CON; age day 35 of ORG).

### 2.4 Blood Sample Collection

Blood samples (~5 mL) were drawn at suckling, one week after suckling (pre‐weaning), and one day after weaning (days 21, 28, 29 of CON; days 35, 42, 43 of ORG). Blood samples were collected through venepuncture of the jugular vein into pre‐labelled tubes lined with K3‐EDTA anticoagulant for haematology analysis. Two piglets per litter (a male and a female) were euthanised via electronarcosis and subsequent exsanguination at 10 weeks of life (day 70). Blood with K3‐EDTA anticoagulant was collected for downstream haematology analysis.

### 2.5 Haematological Analyses

For each blood sampling point, a complete blood count (CBC) was performed to assess the pigs' haematological profiles. The individual blood samples were analysed using an automated veterinary haematology analyser (IDEXX ProCyte Dx, IDEXX Laboratories Inc., Westbrook, Maine, USA) with a species‐specific setting for pigs in the multi‐species software developed by the manufacturer. Approximately 100 μL of whole blood collected in K₃‐EDTA tubes was used for haematology. Reported haematological parameters include: total red blood cell count (RBC, M/μL), haemoglobin concentration (HGB, g/dL), haematocrit (HCT, %), mean corpuscular volume (MCV, fL), mean corpuscular haemoglobin (MCH, pg), mean corpuscular haemoglobin concentration (MCHC, g/dL), total white blood cell count (WBC, K/μL), absolute counts of neutrophils (NEUT, K/μL), lymphocytes (LYMPH, K/μL), monocytes (MONO, K/μL), eosinophils (EO, K/μL), basophils (BASO, K/μL), platelet count (PLT, K/μL) and mean platelet volume (MPV, fL).

### 2.6 Statistical Analyses

All statistical analyses were carried out using R Statistical Software (R Core Team, v4.4.2, Vienna, Austria). Prior to statistical testing, outliers for each blood parameter at each time point were identified using the Grubbs' Test (*p* < 0.01; R package ‘outliers’ v0.15), and subsequently removed from the dataset. Following outlier removal, a Kruskal‐Wallis H‐test was conducted to assess the main effects of breed, housing condition, timepoints and the interaction between breed × housing (R package ‘stats’). Respective contrasts between breeds within each housing condition per timepoint were considered (*p* < 0.05). Notably, due to differing sampling schedules between housing conditions (i.e., different ages at sampling), comparisons involving interactions between housing and time points were not conducted at suckling, pre‐weaning and weaning timepoints. For data observed at slaughter (day 70) as well as data referring to the difference between weaning and pre‐weaning (delta‐change), the same statistical approach was used contrasting breed and housing conditions. Pairwise Spearman correlation coefficients were calculated between all blood parameters, and significance was determined using false discovery rate (FDR)‐corrected *p*‐values. Only correlations with FDR‐adjusted *p* < 0.05 were visualised using R (R package ‘ggplot2’, v3.5.1). For longitudinal analysis, the data was further used in mixed‐effect models considering repeated measurements, as all animals were sampled at multiple time points throughout the study (R package ‘stats’). The model was applied for each housing condition and included breed, timepoint, and the interaction of breed × timepoint as fixed effects as well as the individual pig as a random effect. Time series plots were generated by calculating the mean and standard error of the mean (SEM) for each blood parameter within each experimental group and timepoint, and visualised using line plots with SEM error bars (GraphPad Prism v8.4.3 for Windows, GraphPad Software, Boston, MA, USA).

## 3 Results

### 3.1 Breed Effects on Erythrocyte‐Related Blood Parameters

The GS piglets showed significantly higher RBC counts compared to GL pigs across developmental timepoints, with 8.8%, 9.0%, 8.2% and 12.3% higher RBC counts at suckling (Table 1), pre‐weaning (Table 2), weaning (Table 3) and slaughter (Table 4). For HGB, significant breed differences were only found in CON housing at the time of weaning (GS<GL) and at slaughter (GS>GL). No significant breed effects were observed for HCT. Mean corpuscular volume (MCV) and mean corpuscular haemoglobin (MCH) were significantly higher at all developmental stages in GL pigs compared to GS pigs across both housing systems. With respect to mean corpuscular haemoglobin concentration (MCHC), at suckling, pre‐weaning and weaning, GL pigs under CON housing exhibited significantly higher values than their GS counterparts. However, at slaughter, this pattern shifted and the GS breed was observed to have statistically significantly higher MCHC values compared to GL. Notably, no significant changes in MCHC were observed under ORG housing, regardless of the stage investigated.

**TABLE 1:** Haematological parameters during suckling in German Landrace (GL) and German Saddleback (GS) piglets raised under conventional (CON) and organic (ORG) housing conditions (mean ± SD).

Parameter	CON (day 21)		ORG (day 35)		*p*	
	GS	GL	GS	GL	CON: GS vs. GL	ORG: GS vs. GL
	(*N* = 116)	(*N* = 143)	(*N* = 110)	(*N* = 130)		
Erythrocyte‐related blood parameters						
RBC (M/μL)	6.57 ± 0.65	5.93 ± 0.80	7.11 ± 0.62	6.64 ± 0.62	< 0.001	< 0.001
HGB (g/dL)	12.13 ± 1.18	12.18 ± 1.29	11.33 ± 1.42	11.35 ± 1.21	ns	ns
HCT (%)	41.09 ± 4.50	40.46 ± 4.07	36.04 ± 4.72	36.01 ± 4.14	ns	ns
MCV (fL)	62.65 ± 5.07	68.91 ± 6.91	50.46 ± 4.65	54.26 ± 3.95	< 0.001	0.001
MCH (pg)	18.52 ± 1.47	20.71 ± 1.87	15.88 ± 1.38	17.13 ± 1.32	< 0.001	< 0.001
MCHC (g/dL)	29.60 ± 1.38	30.12 ± 1.41	31.51 ± 1.58	31.58 ± 1.33	0.030	ns
Leukocyte‐related blood parameters						
WBC (K/μL)	12.74 ± 3.39	11.29 ± 3.55	18.78 ± 4.34	15.38 ± 3.87	0.013	< 0.001
NEUT (K/μL)	4.04 ± 1.59	4.16 ± 1.94	5.96 ± 2.1	5.71 ± 2.04	ns	ns
LYMPH (K/μL)	7.59 ± 2.38	6.09 ± 1.85	11.07 ± 2.77	8.37 ± 2.01	< 0.001	< 0.001
MONO (K/μL)	0.96 ± 0.37	0.83 ± 0.35	1.46 ± 0.52	1.06 ± 0.51	0.027	< 0.001
EO (K/μL)	0.107 ± 0.07	0.097 ± 0.062	0.147 ± 0.098	0.139 ± 0.09	ns	ns
BASO (K/μL)	0.009 ± 0.008	0.010 ± 0.01	0.017 ± 0.013	0.020 ± 0.013	ns	ns
Platelet parameters						
PLT (K/μL)	537.3 ± 136.9	529.5 ± 128.3	552.6 ± 159.8	671.1 ± 172.9	ns	< 0.001
MPV (fL)	11.21 ± 1.31	9.19 ± 1.17	11.65 ± 1.46	9.27 ± 1.76	< 0.001	< 0.001

*Note:* Significant differences were considered at *p* < 0.05. Abbreviations: BASO, basophil count; EO, eosinophil count; HCT, haematocrit; HGB, haemoglobin; LYMPH, lymphocyte count; MCH, mean corpuscular haemoglobin; MCHC, mean corpuscular haemoglobin concentration; MCV, mean corpuscular volume; MONO, monocyte count; MPV, mean platelet volume; NEUT, neutrophil count; ns, not significant; PLT, platelet count; RBC, red blood cell count; WBC, white blood cell count.

**TABLE 2:** Haematological parameters at pre‐weaning in German Landrace (GL) and German Saddleback (GS) piglets raised under conventional (CON) and organic (ORG) housing conditions (mean ± SD).

Parameter	CON (day 27)		ORG (day 41)		*p*	
	GS	GL	GS	GL	CON: GS vs. GL	ORG: GS vs. GL
	(*N* = 89)	(*N* = 138)	(*N* = 94)	(*N* = 110)		
Erythrocyte‐related blood parameters						
RBC (M/μL)	7.08 ± 0.52	6.42 ± 0.66	7.17 ± 0.59	6.65 ± 0.67	< 0.001	< 0.001
HGB (g/dL)	12.30 ± 0.96	12.44 ± 1.12	10.79 ± 1.20	10.93 ± 1.22	ns	ns
HCT (%)	40.77 ± 3.55	40.40 ± 3.96	34.51 ± 4.11	34.85 ± 4.46	ns	ns
MCV (fL)	57.66 ± 4.52	63.48 ± 5.52	48.21 ± 5.10	52.28 ± 4.80	< 0.001	0.001
MCH (pg)	17.40 ± 1.22	19.57 ± 1.67	15.10 ± 1.58	16.43 ± 1.49	< 0.001	< 0.001
MCHC (g/dL)	30.23 ± 1.34	30.86 ± 1.30	31.28 ± 1.22	31.38 ± 1.22	0.005	ns
Leukocyte‐related blood parameters						
WBC (K/μL)	16.25 ± 4.53	13.80 ± 3.68	19.36 ± 4.24	16.31 ± 3.45	< 0.001	< 0.001
NEUT (K/μL)	4.99 ± 2.13	5.07 ± 2.07	6.24 ± 2.39	5.85 ± 1.99	ns	ns
LYMPH (K/μL)	9.49 ± 2.92	7.48 ± 1.81	11.48 ± 2.71	9.19 ± 2.04	< 0.001	< 0.001
MONO (K/μL)	1.46 ± 0.59	0.96 ± 0.34	1.47 ± 0.54	1.09 ± 0.45	< 0.001	< 0.001
EO (K/μL)	0.122 ± 0.088	0.111 ± 0.068	0.150 ± 0.087	0.157 ± 0.087	ns	ns
BASO (K/μL)	0.013 ± 0.012	0.014 ± 0.011	0.020 ± 0.015	0.018 ± 0.014	ns	ns
Platelet parameters						
PLT (K/μL)	538.3 ± 131.7	569.0 ± 136.2	581.2 ± 177.0	682.7 ± 152.0	ns	< 0.001
MPV (fL)	11.09 ± 1.56	8.59 ± 0.9	11.98 ± 1.18	8.69 ± 1.60	< 0.001	< 0.001

*Note:* Significant differences were considered at *p* < 0.05. Abbreviations: BASO, basophil count; EO, eosinophil count; HCT, haematocrit; HGB, haemoglobin; LYMPH, lymphocyte count; MCH, mean corpuscular haemoglobin; MCHC, mean corpuscular haemoglobin concentration; MCV, mean corpuscular volume; MONO, monocyte count; MPV, mean platelet volume; NEUT, neutrophil count; ns, not significant; PLT, platelet count; RBC, red blood cell count; WBC, white blood cell count.

**TABLE 3:** Haematological parameters at weaning in German Landrace (GL) and German Saddleback (GS) piglets raised under conventional (CON) and organic (ORG) housing conditions (mean ± SD).

Parameter	CON (day 29)		ORG (day 43)		*p*	
	GS	GL	GS	GL	CON: GS vs. GL	ORG: GS vs. GL
	(*N* = 90)	(*N* = 139)	(*N* = 94)	(*N* = 111)		
Erythrocyte‐related blood parameters						
RBC (M/μL)	6.80 ± 0.64	6.34 ± 0.64	7.17 ± 0.60	6.57 ± 0.61	< 0.001	< 0.001
HGB (g/dL)	11.52 ± 1.27	12.04 ± 1.11	10.56 ± 1.16	10.63 ± 1.09	0.019	ns
HCT (%)	38.74 ± 4.24	39.64 ± 3.87	34.68 ± 4.09	34.66 ± 3.83	ns	ns
MCV (fL)	57.04 ± 4.40	62.85 ± 5.53	48.51 ± 5.49	52.89 ± 4.74	< 0.001	< 0.001
MCH (pg)	16.94 ± 1.20	19.09 ± 1.63	14.79 ± 1.68	16.23 ± 1.55	< 0.001	< 0.001
MCHC (g/dL)	29.74 ± 1.07	30.40 ± 1.12	30.45 ± 1.11	30.70 ± 1.12	< 0.001	ns
Leukocyte‐related blood parameters						
WBC (K/μL)	16.84 ± 4.34	14.79 ± 4.20	20.19 ± 4.54	18.21 ± 3.75	0.002	0.022
NEUT (K/μL)	6.52 ± 3.06	6.17 ± 2.80	7.24 ± 2.76	7.66 ± 2.74	ns	ns
LYMPH (K/μL)	8.63 ± 2.46	7.22 ± 1.89	10.87 ± 2.43	9.05 ± 1.64	< 0.001	< 0.001
MONO (K/μL)	1.55 ± 0.49	1.14 ± 0.49	1.64 ± 0.61	1.27 ± 0.49	< 0.001	< 0.001
EO (K/μL)	0.122 ± 0.089	0.140 ± 0.090	0.208 ± 0.120	0.218 ± 0.116	ns	ns
BASO (K/μL)	0.012 ± 0.012	0.012 ± 0.011	0.016 ± 0.010	0.018 ± 0.012	ns	ns
Platelet parameters						
PLT (K/μL)	535.0 ± 134.2	134.2 ± 139.2	575.0 ± 189.8	668.2 ± 163.0	ns	< 0.001
MPV (fL)	10.48 ± 1.88	8.26 ± 0.86	11.88 ± 1.35	8.52 ± 1.46	< 0.001	< 0.001

*Note:* Significant differences were considered at *p* < 0.05. Abbreviations: BASO, basophil count; EO, eosinophil count; HCT, haematocrit; HGB, haemoglobin; LYMPH, lymphocyte count; MCH, mean corpuscular haemoglobin; MCHC, mean corpuscular haemoglobin concentration; MCV, mean corpuscular volume; MONO, monocyte count; MPV, mean platelet volume; NEUT, neutrophil count; ns, not significant; PLT, platelet count; RBC, red blood cell count; WBC, white blood cell count.

**TABLE 4:** Haematological parameters at day 70 (slaughter) in German Landrace (GL) and German Saddleback (GS) pigs raised under conventional (CON) and organic (ORG) housing conditions (mean ± SD).

Parameter	CON (day 70)		ORG (day 70)		*p*			
	GS	GL	GS	GL	CON: GS vs. GL	ORG: GS vs. GL	GS: CON vs. ORG	GL: CON vs. ORG
	(*N* = 38)	(*N* = 52)	(*N* = 34)	(*N* = 40)				
Erythrocyte‐related blood parameters								
RBC(M/μL)	7.84 ± 0.66	7.04 ± 0.67	7.99 ± 0.74	7.05 ± 0.72	< 0.001	< 0.001	ns	ns
HGB (g/dL)	12.56 ± 1.35	11.95 ± 1.08	12.24 ± 1.11	11.58 ± 1.19	0.040	ns	ns	ns
HCT (%)	40.74 ± 4.06	39.72 ± 4.09	38.41 ± 3.35	37.02 ± 3.78	ns	ns	0.037	0.011
MCV (fL)	51.96 ± 2.52	56.49 ± 3.76	48.22 ± 3.55	52.65 ± 3.33	< 0.001	< 0.001	0.002	< 0.001
MCH (pg)	16.00 ± 0.84	17.01 ± 1.15	15.38 ± 1.22	16.47 ± 1.06	< 0.001	< 0.001	ns	ns
MCHC (g/dL)	30.82 ± 0.92	30.13 ± 0.96	31.77 ± 1.48	31.28 ± 0.91	0.017	ns	0.012	< 0.001
Leukocyte‐related blood parameters								
WBC (K/μL)	22.17 ± 5.64	19.38 ± 5.34	24.52 ± 6.28	20.29 ± 5.83	0.039	0.014	ns	ns
NEUT (K/μL)	10.24 ± 3.61	9.27 ± 4.19	11.43 ± 3.81	8.98 ± 3.36	ns	0.018	ns	ns
LYMPH (K/μL)	9.98 ± 2.83	8.92 ± 2.34	11.54 ± 3.14	9.94 ± 3.15	ns	ns	ns	ns
MONO (K/μL)	1.41 ± 0.44	1.08 ± 0.42	1.45 ± 0.57	1.19 ± 0.57	0.003	ns	ns	ns
EO (K/μL)	0.071 ± 0.050	0.078 ± 0.073	0.084 ± 0.085	0.082 ± 0.080	ns	ns	ns	ns
BASO (K/μL)	0.018 ± 0.011	0.016 ± 0.009	0.017 ± 0.01	0.018 ± 0.011	ns	ns	ns	ns
Platelet parameters								
PLT (K/μL)	399.9 ± 78.2	426.5 ± 104.6	413.8 ± 121.1	517.3 ± 89	ns	< 0.001	ns	< 0.001
MPV (fL)	10.01 ± 2.87	7.81 ± 0.63	13.55 ± 1.86	8.44 ± 1.90	ns	< 0.001	ns	ns

*Note:* Significant differences were considered at *p* < 0.05. Abbreviations: BASO, basophil count; EO, eosinophil count; HCT, haematocrit; HGB, haemoglobin; LYMPH, lymphocyte count; MCH, mean corpuscular haemoglobin; MCHC, mean corpuscular haemoglobin concentration; MCV, mean corpuscular volume; MONO, monocyte count; MPV, mean platelet volume; NEUT, neutrophil count; ns, not significant; PLT, platelet count; RBC, red blood cell count; WBC, white blood cell count.

### 3.2 Breed Effects on Leukocyte‐Related Blood Parameters

Across all developmental stages and housing conditions (Tables 1, 2, 3, 4), GS pigs had higher total white blood cell (WBC) counts than GL pigs. GS pigs housed in ORG conditions exhibited 27.3% higher NEUT counts than their GL counterparts at slaughter. At earlier developmental stages and CON housing, NEUT counts did not differ. For LYMPH, significant breed differences were observed at the time of suckling, pre‐weaning, and weaning under both housing conditions (GS>GL). Similarly, GS pigs showed elevated MONO counts compared to GL pigs in CON and ORG across all developmental timepoints, with the exception of ORG housing at slaughter, in which the breed effect was insignificant. No significant effects were observed for EO and BASO.

### 3.3 Breed Effects on Platelet Parameters

With respect to PLT, GL pigs under ORG housing exhibited significantly higher values than their GS counterpart with 21.4%, 17.5%, 16.2% and 25.0% higher values at suckling (Table 1), pre‐weaning (Table 2), weaning (Table 3) and slaughter (Table 4) exclusively. The GS pigs showed significantly higher MPV concentrations compared to their GL counterparts in both husbandry conditions at the time of suckling, pre‐weaning and weaning. Interestingly, at slaughter, GS pigs also showed statistically significant elevated MPV concentrations compared to GL pigs, but this was observed only under ORG housing conditions.

### 3.4 Housing Effects on Blood Count Parameters at Slaughter

No significant housing effects were observed for RBC and HGB (Table 4). Both breeds under CON housing showed significantly higher percentages of HCT (7.3% and 6.1%) and MCV (7.3% and 7.8%) counts respectively compared to ORG housing (GL‐CON>GL‐ORG & GS‐CON>GS‐ORG). No significant housing effects were observed for MCH at slaughter. With respect to MCHC, both breeds under organic housing showed significantly higher counts compared to conventional housing (GL‐CON<GL‐ORG & GS‐CON<GS‐ORG). No significant housing effects were observed for LYMPH and MONO. The GL pigs under ORG housing showed higher PLT counts compared to their GL counterparts housed in the CON system. No significant housing effects were observed for MPV at slaughter.

**FIGURE 1:**
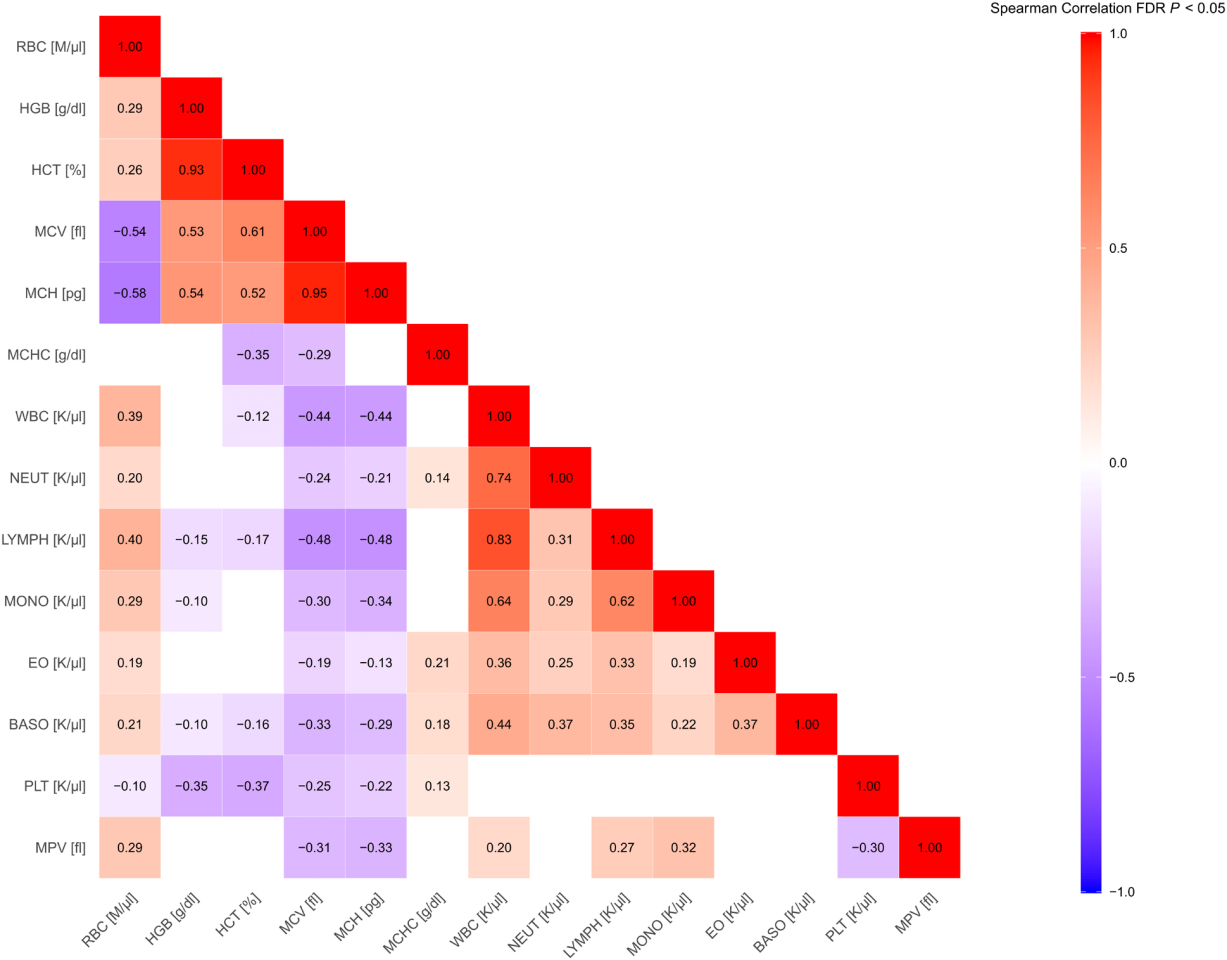
Phenotypic correlation heatmap of haematological parameters in pigs. The heatmap represents pairwise Spearman correlation coefficients (ρ) between 14 blood parameters, visualized as a colour gradient from strong negative (blue) to strong positive (red) correlations. Only statistically significant FDR‐corrected correlations are shown (adjusted *p* < 0.05). BASO, basophil count; EO, eosinophil count; HCT, haematocrit; HGB, haemoglobin; LYMPH, lymphocyte count; MCH, mean corpuscular haemoglobin; MCHC, mean corpuscular haemoglobin concentration; MCV, mean corpuscular volume; MONO, monocyte count; MPV, mean platelet volume; NEUT, neutrophil count; PLT, platelet count; RBC, red blood cell count; WBC, white blood cell count.

All reported Spearman correlations were statistically significant, unless otherwise noted (Figure 1). Strong positive correlations were identified between related erythrocyte indices: HGB and HCT (ρ = 0.93), and MCV and MCH (ρ = 0.95). Interestingly, RBC counts showed strong negative correlations with MCV (ρ = −0.54) and MCH (ρ = −0.58), suggestive of smaller, less haemoglobin‐loaded cells. Additionally, HGB displayed moderate positive correlations with erythrocyte size indices such as MCV (ρ = 0.53), indicative that haemoglobin content may be following cell size more closely than cell number. With respect to leukocytes and its subsets, lymphocytes and neutrophils comprised most of the leukocyte variability as WBC vs. LYMPH and WBC vs. NEUT had strong positive correlations of ρ = 0.83 and ρ = 0.74, respectively. Monocyte counts also followed WBC closely (ρ = 0.64), whereas correlations of WBC to EO (ρ = 0.36) and BASO (ρ = 0.44) were weaker, but still positive. The correlation between NEUT and LYMPH was positive (ρ = 0.31). With respect to platelet parameters, larger platelets compensated for lower platelet numbers, with PLT counts inversely correlating with MPV (ρ = −0.30). MPV also showed weak positive correlations with several leukocyte parameters (e.g., MPV vs. MONO, ρ = 0.32).

### 3.5 Longitudinal Analysis of Erythrocyte‐Related Blood Parameters

**FIGURE 2:**
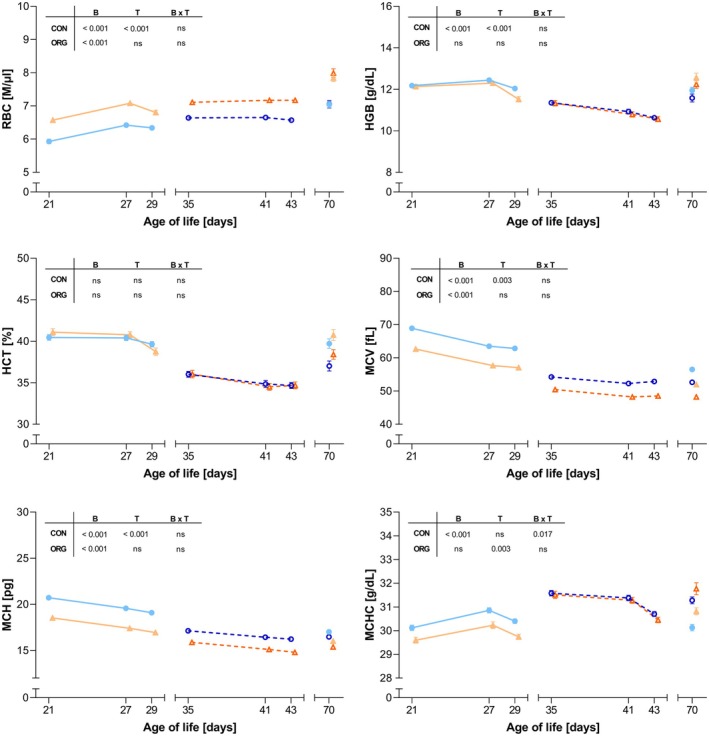
Time‐course analysis of erythrocyte profiles in GL (German Landrace) and GS (German Saddleback) pigs under CON (conventional) and ORG (organic) housing systems. The plots show longitudinal changes in erythrocyte parameters from 21 to 70 days of age with GL (blue lines) and GS (orange lines) housed in either CON (solid line, triangles) or ORG (dashed lines, squares). Each data point represents the mean value. Error bars are representative of the standard error of the mean (SEM). The inserted tables within each panel represent the resulting *p*‐values (*p* < 0.05; ns: Not significant) for the main effects of breed (B), time (T) and the breed × time interaction (B × T). HCT, haematocrit; HGB, haemoglobin; MCH, mean corpuscular haemoglobin; MCHC, mean corpuscular haemoglobin concentration; MCV, mean corpuscular volume; RBC, red blood cell count.

Time‐series analyses of erythrocytes indicated significant breed effects on RBC, MCV and MCH across both housing conditions as well as breed effects on HGB and MCHC in CON housing (Figure 2). In CON housing, with a longer weaning‐to‐slaughter interval compared to ORG, the time effect was significant for RBC, HGB, MCV and MCH. For MCHC, time significantly affected ORG‐housed animals, while CON‐housed animals showed a significant breed × time interaction. Notably, MCHC declined post‐weaning in both housing systems. No significant effects were observed for HCT. Comparison of data stating pre‐weaning (CON: day 27; ORG: day 41) and post‐weaning (CON: day 29; ORG: day 43) measurements are shown in Table 5. Following weaning, in CON housing, RBC, HGB and HCT showed a significant decline in GS (4.1%, 6.8% and 5.2%) compared to GL piglets (1.3%, 3.3% and 1.9%) respectively, while in ORG no breed differences were observed. With the exception of RBC in GL and MCHC in both breeds, there were significant differences between housing conditions within the breed, with CON showing a larger decline in these parameters compared to ORG (Table 5).

**TABLE 5:** Change in haematological parameters in response to weaning in German Landrace (GL) and German Saddleback (GS) pigs raised under conventional (CON) and organic (ORG) housing. The delta change was calculated as the difference between measurements taken immediately after weaning (day 29 for CON, day 43 for ORG) and pre‐weaning (day 27 for CON, day 41 for ORG).

Parameter	CON		ORG		*p*			
	GS	GL	GS	GL	CON: GS vs. GL	ORG: GS vs. GL	GS: CON vs. ORG	GL: CON vs. ORG
Erythrocyte‐related blood parameters								
RBC (M/μL)	−0.29 ± 0.50	−0.08 ± 0.34	0.00 ± 0.44	−0.08 ± 0.46	0.006	ns	< 0.001	ns
HGB (g/dL)	−0.79 ± 0.83	−0.41 ± 0.73	−0.23 ± 0.62	−0.30 ± 0.91	0.003	ns	< 0.001	0.043
HCT (%)	−2.07 ± 2.95	−0.76 ± 2.83	0.17 ± 2.72	−0.19 ± 3.49	0.024	ns	< 0.001	0.023
MCV (fL)	−0.65 ± 2.38	−0.63 ± 2.23	0.29 ± 1.30	0.61 ± 1.70	ns	ns	0.006	< 0.001
MCH (pg)	−0.46 ± 0.37	−0.48 ± 0.41	−0.31 ± 0.26	−0.19 ± 0.39	ns	ns	0.003	< 0.001
MCHC (g/dL)	−0.48 ± 1.20	−0.46 ± 1.19	−0.83 ± 0.99	−0.70 ± 0.89	ns	ns	ns	ns
Leukocyte‐related blood parameters								
WBC (K/μL)	0.55 ± 4.18	1.00 ± 3.61	0.83 ± 4.12	1.90 ± 3.54	ns	0.031	ns	ns
NEUT (K/μL)	1.53 ± 2.56	1.04 ± 2.25	1.03 ± 2.21	1.81 ± 2.55	ns	ns	ns	0.023
LYMPH (K/μL)	−0.97 ± 2.29	−0.26 ± 1.72	−0.52 ± 2.07	−0.14 ± 1.52	ns	ns	ns	ns
MONO (K/μL)	0.09 ± 0.54	0.17 ± 0.42	0.17 ± 0.57	0.18 ± 0.45	ns	ns	ns	ns
EO (K/μL)	0.002 ± 0.077	0.030 ± 0.079	0.060 ± 0.095	0.059 ± 0.093	0.011	ns	< 0.001	0.022
BASO (K/μL)	−0.001 ± 0.015	−0.002 ± 0.012	−0.004 ± 0.012	0.000 ± 0.016	ns	ns	ns	ns
Platelet parameters								
PLT (K/μL)	−3.1 ± 107.7	−7.3 ± 133.7	−16.4 ± 106.4	−14.5 ± 105.3	ns	ns	ns	ns
MPV (fL)	−0.12 ± 0.40	−0.31 ± 0.61	−0.01 ± 0.49	−0.04 ± 0.40	0.028	ns	ns	< 0.001

*Note:* Data are presented as mean ± standard deviation (SD). Significant differences were considered at *p* < 0.05. Abbreviations: BASO, basophil count; EO, eosinophil count; HCT, haematocrit; HGB, haemoglobin; LYMPH, lymphocyte count; MCH, mean corpuscular haemoglobin; MCHC, mean corpuscular haemoglobin concentration; MCV, mean corpuscular volume; MONO, monocyte count; MPV, mean platelet volume; NEUT, neutrophil count; ns, not significant; PLT, platelet count; RBC, red blood cell count; WBC, white blood cell count.

### 3.6 Longitudinal Analysis of Leukocyte‐Related Blood Parameters

**FIGURE 3:**
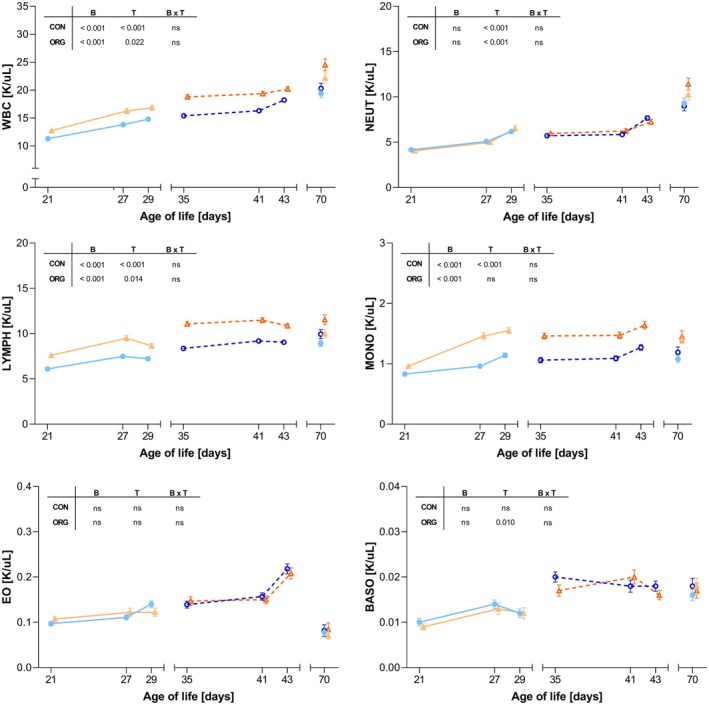
Time‐course analysis of leukocyte profiles in GL (German Landrace) and GS (German Saddleback) pigs under CON (conventional) and ORG (organic) housing systems. The plots show longitudinal changes in leukocyte parameters from 21 to 70 days of age with GL (blue lines) and GS (orange lines), housed in either CON (solid line, triangles) or ORG (dashed lines, squares). Each data point represents the mean value. Error bars are representative of the standard error of the mean (SEM). The inserted tables within each panel represent the resulting *p*‐values (*p* < 0.05; ns: Not significant) for the main effects of breed (B), time (T) and the breed × time interaction (B × T). BASO, basophil count; EO, eosinophil count; LYMPH, lymphocyte count; MONO, monocyte count; NEUT, neutrophil count; WBC, white blood cell count.

The GS pigs showed higher WBC counts compared to GL pigs across all developmental ages where the breed and time effects were significant under both housing conditions (Figure 3). The same differences were identified for LYMPH and MONO, with the exception that for MONO, no time effect was found under ORG housing. NEUT counts increased under both housing conditions over time, with the profiles showing an overlap for the two breeds. No significant breed and time effects were observed for EO, while under ORG housing, a significant time effect was observed for BASO counts. No significant effect of breed × time interaction was observed for the leukocyte parameters. For leukocytes, weaning induced significant increases in total WBC under ORG housing in GL (11.7%) compared to GS (4.3%) (Table 5). The change in NEUT counts due to weaning was also significantly affected by housing system, specifically with GL pigs exhibiting a 42.5% increase in mean counts under ORG compared to CON. No significant effects were observed for LYMPH, MONO and BASO. GL piglets housed in ORG displayed significantly higher increases in EO counts than their counterparts under CON. Additionally, the change in EO counts differed significantly between housing systems where piglets in ORG consistently maintained higher counts following weaning than piglets under CON housing.

### 3.7 Longitudinal Analysis of Platelet Parameters

**FIGURE 4:**
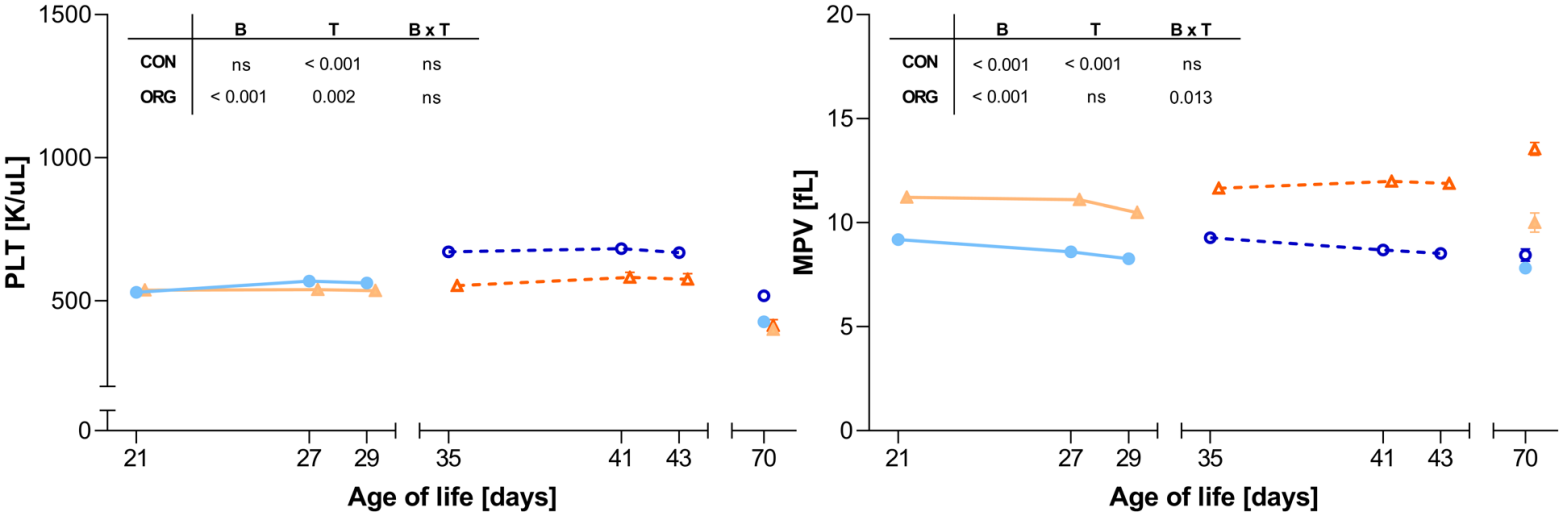
Time‐course analysis of platelet profiles in GL (German Landrace) and GS (German Saddleback) pigs under CON (conventional) and ORG (organic) housing systems. The plots show longitudinal changes in platelet parameters from 21 to 70 days of age with GL (blue lines) and GS (orange lines) housed in either CON (solid line, triangles) or ORG (dashed lines, squares). Each data point represents the mean value. Error bars are representative of the standard error of the mean (SEM). The inserted tables within each panel represent the resulting *p*‐values (*p* < 0.05; ns: Not significant) for the main effects of breed (B), time (T) and the breed × time interaction (B × T). MPV, mean platelet volume; PLT, platelet count.

In ORG housing, PLT levels were significantly affected by breed, with GL pigs exhibiting higher PLT than GS pigs (Figure 4). Furthermore, a time‐dependent reduction in trunk blood PLT at slaughter was observed in both CON and ORG housing. With respect to MPV, a significant effect of breed was observed under both CON and ORG, with GS pigs showing notably higher MPV values compared to GL pigs. In addition, a time effect was revealed for CON housing and the interaction of breed × time was found to be statistically significant under the ORG system. Concerning the effect of weaning on platelet parameters (Table 5), no significant effects were observed for PLT. Within CON housing, GL piglets experienced a significantly greater decrease in MPV compared to GS. In addition, the greater reduction in GL‐CON piglets was also observed for GL‐ORG.

## 4 Discussion

Breed‐specific differences in haematological parameters were observed throughout early development of pigs in which both breeds exhibited differing erythrocyte and leukocyte profiles that reflect their unique selection histories and physiological demands. Our findings address developmental, breed and housing effects in the context of oxygen delivery capacity, immune resilience and the nutritional and environmental conditions imposed by conventional and organic management systems.

### 4.1 Breed and Housing Effects Within Physiological Reference Intervals

While statistically significant breed differences were observed in key erythrocyte and leukocyte parameters, it is essential to compare these findings against established reference intervals for piglets of similar ages. Although GS piglets in our study consistently exhibited higher RBC, WBC, LYMPH and MONO counts across the developmental periods, these observed values remained within the 95% reference intervals (RI) reported for healthy piglets of similar ages. For instance, RIs that were reported for crossbred nursery pigs: RBC 5.62–7.84 (10^12^/L), WBC 7.18–24.52 (10^9^/L), LYMPH 1.84–12.42 (10^9^/L) and MONO 0.25–2.81 (10^9^/L); and similarly for Ontario piglets: RBC 4.8–7.3 (10^12^/L) and WBC 6.0–21.7 (10^9^/L) (Zhang et al. 2022; Perri et al. 2017). Reference intervals for the GS are, however, scarce. A recent study confirms that traditional breeds often maintain higher baseline leukocyte and lymphocyte counts compared to modern commercial lines (Stevančević et al. 2025). The observed differences between GS and GL in our study substantiate breed‐specific physiological reference intervals, which are influenced by their respective selection history and specific requirements.

### 4.2 Breed and Housing Effects on Erythrocyte Characteristics

Erythrocyte parameters showed statistically significant breed differences, with GS pigs exhibiting higher RBC counts than GL pigs, irrespective of age and housing condition. Parameters reflecting red blood cell size (MCV) and haemoglobin content (MCH) were higher in GL pigs. This demonstrates they possess larger, more haemoglobin‐rich erythrocytes, a characteristic beneficial for rapid growth and muscle accretion (Oven 2025). Further, there was a strong negative correlation found between RBC and MCV. The observed differences in erythrocyte parameters between breeds are consistent with previous studies, which reported that the native Slovenian Krškopolje breed exhibits approximately 6.74 × 10^12^ RBC/L pointing to a genetic control of erythropoiesis (Sun et al. 2025; Oven 2025). Divergent erythrocyte profiles may therefore be indicative of distinct capacities for delivering oxygen to muscle tissue with potential impacts on metabolic rate and muscle function as reported for several other mammals (Lindholm‐Perry et al. 2021). Indeed, breed‐specificities have been reported at phenotypic levels such as myoglobin content and muscle fibre distribution in *M. longissimus dorsi* (Gao et al. 2024; Wimmers et al. 2008). Specifically, higher myoglobin concentrations were revealed in breeds like Chester White (0.92 mg/g), Hampshire (0.95 mg/g) and Duroc (0.85 mg/g) compared to Landrace (0.62 mg/g). Higher RBC counts and smaller cell size maximise oxygen uptake, largely through increasing the absolute erythrocyte surface area for gas exchange (Storz 2021). Whereas larger and more haemoglobin‐rich erythrocytes may support intensive metabolic demand, thus reflecting breed characteristics in the adaptation to different environments, management practices and physiological demands.

The GL piglets in the CON housing system maintained significantly higher MCHC level than their GS‐CON counterparts during all stages of development, while no such breed effect was found under ORG conditions. Given that iron is crucial for haemoglobin synthesis and MCHC reflects haemoglobin concentration per erythrocyte, maintaining high MCHC levels requires efficient iron absorption and utilisation (Szudzik et al. 2018). The sustained breed‐specific descriptive observations of MCHC levels under CON are consistent with the hypothesis that commercial pigs, selected for rapid growth and muscle accretion, potentially exhibit a more efficient iron utilisation to match the high erythropoietic demands in CON. This is particularly critical during the early postnatal and weaning stages, as high growth rates of commercial piglets necessitate a rapid expansion of blood volume that can quickly deplete neonatal iron stores (Lipiński et al. 2026). While direct measures of iron status were not performed, this observation aligns with established breed‐specific variations in iron metabolism (Szudzik et al. 2018). Recent data indicate that breed and management dependent differences in blood parameters are already evident at birth. In CON housing, neonatal GS piglets exhibited higher MCHC within the first hours of life compared to GL piglets (Oster et al. 2026), which is in contrast with the findings in this study from day 21 onwards. The reversal in MCHC levels under CON conditions suggests that early advantages in iron availability or erythropoietic status in indigenous pig breeds may be gradually offset by improved postnatal iron absorption efficiency and erythropoietic capacity in commercial breeds. Recent studies have shown that indigenous breeds may be more sensitive to indoor housing where they lack access to soil‐based iron (Prunier et al. 2022). Similar to the observations in this study, Oven (2025) has suggested that organic management may lead to significantly higher MCHC levels compared to conventional systems, potentially due to the ingestion of environmental iron. This suggests that while the GL breed may be optimised for the intensive management within CON, ORG housing may mitigate the physiological difference between commercial and traditional breeds (Prunier et al. 2022). Collectively, these findings reflect a trade‐off between erythrocyte quantity and haemoglobin‐loading capacity, with each breed adopting a distinct oxygen delivery strategy consistent with its respective selection history, though both remain sensitive to the iron availability imposed by the housing environment.

### 4.3 Immune Resilience Assessed From Leukocyte and Platelet Profiles

Breed differences were evident within leukocyte profiles, with GS pigs exhibiting markedly elevated WBC, LYMPH and MONO counts compared to GL pigs across all developmental stages and housing conditions. Similar results have been reported previously, where indigenous breeds like the Andaman wild pig and the Nicobari pig displayed significantly higher total leukocytes than the commercially selected Large White Yorkshire (De et al. 2013). Elevated baseline levels of blood leukocytes may be associated with enhanced immunocompetence, thereby enabling immediate response to pathogens (Radulovic et al. 2022). Via transcriptomic approaches, Yang et al. (2022) reported that indigenous Min pigs showed higher abundance of genes involved in pattern recognition receptor signalling and complement regulation compared to Large White pigs, specifically genes such as *SERPING1*. Similarly, indigenous Balkan Turopolje pigs displayed faster viral clearance from serum and a stronger type 1 (cell‐mediated) immune profile in response to experimental PRRSV vaccination, including higher post‐vaccination leukocyte counts compared to crossbred Landrace × Pietrain pigs (Ballweg et al. 2016). The breed‐specific resilience may also be conferred due to selection pressures in environments with significant pathogen challenge and low‐input, variable husbandry conditions.

Platelet counts were significantly higher in GL piglets compared to GS piglets across all developmental stages under ORG housing, while no breed differences appeared under CON. The observed difference may be consistent with a stronger response to environmental stimuli within the ORG system, though age‐related maturation cannot be ruled out at this stage. Interestingly, a genome‐wide association study identified *BMP6* as a candidate gene affecting platelet count in GL (Ponsuksili et al. 2016). *BMP6* acts as a key endogenous regulator of hepcidin, the principal hormone controlling systemic iron homeostasis (Fisher and Babitt 2022). Breed differences in thrombopoietin sensitivity, growth rate, and iron demand may amplify this effect in GL piglets, whereas the standardised diet and hygiene in CON likely mask such differences. Other studies have reported elevated PLT counts in commercial breeds such as Yorkshire relative to indigenous breeds like the Mangalitza (Kotosová et al. 2014) and pigs raised in organic farms compared to conventional farms (Oven 2025). While not measured in the current study, platelet production is tightly regulated by thrombopoietin and inflammatory cytokines such as IL‐6, which are upregulated due to immune challenges (Hitchcock et al. 2021). Additionally, in the current study there was an inverse correlation found between PLT and MPV (ρ = −0.30), suggesting a mechanism in which larger platelets compensate for lower PLT counts to optimise clotting efficiency. Consequently, it may be inferred that breed‐specific variation in platelet traits reflects different strategies for responding to immune challenges arising from different housing conditions. Collectively, the elevated leukocyte counts observed in GS pigs, while remaining within physiological ranges, likely reflect long‐term selection in variable, pathogen‐rich environments. The results referring to the platelet dynamics further suggest that these breed‐specific immune strategies are not uniformly expressed but are amplified or masked depending on the specificities of the housing system.

### 4.4 Effects of Housing System and Weaning Age

It is important to note that, due to the study design, piglets in ORG housing were weaned and sampled at later stages than those in the CON system during suckling, pre‐weaning and weaning. To avoid interpreting age‐related physiological changes rather than true environmental factors, valid statistical assessment of housing system effects was conducted exclusively at the slaughter timepoint, where all pigs, regardless of housing condition, were euthanised at 70 days of life. Taking this into consideration, ORG‐raised piglets exhibited significantly lower HCT and MCV, although higher MCHC levels compared to CON‐housed piglets. Interestingly, this housing effect is preceded by a different physiological response to weaning (delta change) in which CON piglets experienced a significant decrease in HCT and MCV, whereas ORG piglets (weaned 14 days later at seven weeks of age) showed stable HCT percentages and a significant increase in MCV levels. This is likely representative of the additional 14 days of piglet suckling under ORG, resulting in a different haematological response to weaning. Nutrient deficiency or imbalance can impair red blood cell development, leading to a lesser and smaller number of erythrocytes (Fu et al. 2025). In fact, efficient erythropoiesis relies on an adequate supply of iron, vitamin B12 and folate, and sulphur‐containing amino acids like methionine (Szudzik et al. 2018). Importantly, Ludwiczak et al. (2021) attributed differences in animal welfare to diet variability and micronutrient deficiencies associated with varying husbandry conditions. While both applied housing systems provided methionine in their respective diets, ORG diets lacked the Methionine‐Equivalent MHA supplementation present in CON feeds (Table S1), which suggests a potential influence of diet composition on methionine availability. This could impact the methyl‐donor capacity necessary for erythrocyte maturation (Yang et al. 2020). Future studies incorporating direct amino acid profiling are needed to confirm the extent of this nutritional influence. Specifically, iron‐methionine complexes have been studied as effective iron oral supplements for neonatal pigs (Fu et al. 2025). With respect to the higher MCHC levels exhibited by ORG‐housed piglets, this may be indicative of a compensatory mechanism in response to the variable conditions found in ORG, whereby the smaller erythrocytes (lower MCV) reported in this study might produce sufficient haemoglobin intracellularly to optimise oxygen delivery efficiency (Yang et al. 2024), in spite of the lower overall volume of erythrocytes.

## 5 Conclusion

The applied 2 × 2 factorial design allowed for investigation of breed‐specific and housing‐related influences on blood count parameters throughout early life in pigs. The GS pigs showed higher leukocyte counts, which potentially confer genetic predisposition for resilience in immune function. Moreover, the evaluation of the red blood profiles revealed a breed‐specific strategy for ensuring efficient oxygen uptake, with GS pigs having a high number of smaller erythrocytes and GL pigs having larger, haemoglobin‐rich erythrocytes. Housing comparisons at day 70 revealed lower HCT and MCV but higher MCHC in ORG‐raised piglets, which might indicate a physiological adaptation to weaning management. Future work should aim to clarify molecular mechanisms underlying gene expression patterns driving baseline immune status to improve pig health and immune resilience.

## Author Contributions

Conceptualisation: E.M., K.W.; Data curation: K.K., M.O., H.R.; Formal analysis: K.K., M.O., K.W.; Funding acquisition: K.W.; Investigation: K.K., M.O., H.R., E.M., K.W.; Methodology: K.K., M.O., H.R., C.C.M., E.M., K.W.; Project administration: E.M., S.P., K.W.; Supervision: K.W.; Visualisation: K.K., M.O., H.R.; Roles/Writing – original draft: K.K.; and Writing – review and editing: M.O., H.R., C.C.M., E.M., S.P., K.W.

## Funding

The European Partnership on Animal Health and Welfare (EUPAHW) is co‐funded by the European Union under the Horizon Europe Programme (Grant Agreement No. 101136346).

## Conflicts of Interest

The authors declare no conflicts of interest.

## Supporting information


**Table S1:** Nutrient composition of the experimental diets fed to sows (lactation and gestation diet) and piglets (pre‐starter, creep feed and fattening stages) under conventional (CON) and organic (ORG) housing conditions.

## Data Availability

The data that support the findings of this study are available on request from the corresponding author. The data are not publicly available due to privacy or ethical restrictions.
