# Supplementary material for: Haematopoietic Traits in Indigenous and Modern Pig Breeds Are Modulated by Housing System and Developmental Stage
Source: Anim Genet. 2026 Apr 16;57:e70103. doi: 10.1002/age.70103 (PMC13087205; doi:10.1002/age.70103)
Supplement: Supplementary file 1 — Table S1: Nutrient composition of the experimental diets fed to sows (lactation and gestation diet) and piglets (pre‐starter, creep feed and fattening stages) under conventional (CON) and organic (ORG) housing conditions. [file AGE-57-0-s001.docx]

**Supplemental online material**

**Supplemental Table S1.** Nutrient composition of the experimental diets fed to sows (lactation and gestation diet) and piglets (pre-starter, creep feed, and fattening stages) under conventional (CON) and organic (ORG) housing conditions.

| Dev. Stage |  | | Pre-starter | | Creep feed | | | 1^st^ Fattening | | 2^nd^ Fattening | | | Lactation diet | | Gestation diet | | |
| --- | --- | --- | --- | --- | --- | --- | --- | --- | --- | --- | --- | --- | --- | --- | --- | --- | --- |
| Item | | Unit | CON | ORG | CON | ORG | CON | | ORG | | CON | ORG | CON | ORG | | CON | ORG |
| Crude Protein | | % | 18.5 | 17.5 | 17.5 | - | 16.7 | | 17.2 | | 15.0 | 16.0 | - | 15.7 | | 14.5 | 13.0 |
| Crude Fat | | % | 5.7 | 5.1 | 4.5 | - | 3.0 | | 4.2 | | 3.0 | 3.8 | - | 5.3 | | 4.5 | 2.8 |
| Crude Fiber | | % | 3.0 | 4.5 | 4.0 | - | 3.8 | | 4.5 | | 4.5 | 5.7 | - | 5.4 | | 7.0 | 7.0 |
| Crude Ash | | % | 5.2 | 4.9 | 5.4 | - | 5.0 | | 5.5 | | 4.5 | 4.6 | - | 6.0 | | 5.5 | 5.8 |
| Calcium | | % | 0.60 | 0.70 | 0.70 | - | 0.85 | | 0.74 | | 0.65 | 0.60 | - | 0.82 | | 0.65 | 0.62 |
| Phosphorus | | % | 0.56 | 0.55 | 0.54 | - | 0.48 | | 0.55 | | 0.4 | 0.55 | - | 0.69 | | 0.45 | 0.52 |
| Lysine | | % | 1.35 | 1.00 | 1.28 | - | 1.20 | | 1.00 | | 0.95 | 0.85 | - | 0.95 | | 0.67 | 0.68 |
| Sodium | | % | 0.25 | 0.22 | 0.25 | - | 0.20 | | 0.24 | | 0.20 | 0.20 | - | 0.25 | | 0.25 | 0.21 |
| M-E MHA | | % | 0.22 | - | 0.17 | - | 0.11 | | - | | 0.03 | - | - | - | | - | - |
| Methionine | | % | 0.55 | 0.3 | 0.41 | - | 0.36 | | 0.28 | | 0.28 | 0.25 | - | 0.26 | | 0.23 | 0.19 |
| Metabolizable energy (pig) | | MJ/kg | - | 13.2 | - | - | - | | 13.0 | | 12.8 | 12.8 | - | 13.0 | | 12.2 | 11.8 |
